# Evaluation of the effectiveness of exercise therapy for irritable bowel syndrome: a systematic review and meta-analysis

**DOI:** 10.3389/fmed.2026.1771521

**Published:** 2026-03-10

**Authors:** Jiali Wu, Shaojie Du, Yanting Sun, Jie Ye, Yangxian Xu

**Affiliations:** 1 Longhua Hospital, Shanghai University of Traditional Chinese Medicine, Shanghai, China; 2 Department of General Surgery II, Longhua Hospital, Shanghai University of Traditional Chinese Medicine, Shanghai, China; 3 Department of Orthopedics and Traumatology, Longhua Hospital, Shanghai University of Traditional Chinese Medicine, Shanghai, China; 4 Coloproctology Department, Longhua Hospital, Shanghai University of Traditional Chinese Medicine, Shanghai, China

**Keywords:** clinical trial, exercise therapy, irritable bowel syndrome, meta-analysis, systematic review

## Abstract

**Objective:**

Irritable bowel syndrome (IBS) is a functional gastrointestinal disorder characterized by abdominal pain, distension, and altered bowel habits that significantly impacts patients’ quality of life and imposes a substantial socioeconomic burden. Traditional treatment options, including antispasmodics and probiotics, are often limited by modest efficacy, variable evidence quality, and challenges with long-term adherence, highlighting the need for alternative non-pharmacological strategies. Exercise has gained attention as a non-pharmacological intervention because of its ability to regulate autonomic function and modulate inflammatory pathways. In this review, we define exercise therapy as a planned, structured, and repetitive physical activity program with specified type, frequency, intensity, and duration.

**Methods:**

The PubMed, Embase, Web of Science, and Ovid databases were searched up to February 17, 2025 for studies that compared exercise therapy with no exercise therapy in IBS. A meta-analysis was conducted, and when heterogeneity was excessive, a sensitivity analysis was performed.

**Results:**

Of 2,142 citations screened, 10 studies that included 437 patients with IBS were selected. The meta-analysis indicated that improvement in the IBS-SSS score was greater in the exercise group IBS than in the control group. However, the effects of exercise intervention on the IBS-QOL measure and anxiety were not statistically significant.

**Conclusion:**

Exercise interventions could alleviate symptoms in patients with IBS, although their impact on quality of life scores and remission of anxiety is unclear. There is no evidence-based consensus on a standardized exercise prescription for IBS. The absence of such a framework may introduce potential confounders, affecting the accuracy of efficacy assessments of quality of life and psychological outcomes. Multicenter randomized controlled trials with a standardized exercise framework are needed to explore the role and mechanisms of exercise therapy in management of IBS.

**Systematic review registration:**

https://www.crd.york.ac.uk/PROSPERO/view/CRD420250478248, identifier PROSPERO (CRD420250478248).

## Highlights

Strengths

- Rigorous methodology following PRISMA guidelines with dual independent review.
- Comprehensive analysis of both symptom severity and quality of life outcomes and performed sensitivity analyses to validate findings.

Limitations

- Significant heterogeneity observed across studies (I^2^ = 84–92%).
- Limited by small sample size (10 studies, 437 participants).
- Unable to assess long-term effects due to short follow-up periods.

## Introduction

Irritable bowel syndrome (IBS) is a functional gastrointestinal disorder with a reported worldwide prevalence of up to 5–10% (1). It is characterized primarily by abdominal pain, distension, and altered bowel habits, which lead to a significant decrease in patients’ quality of life and a substantial socioeconomic burden (2–4). Notably, psychiatric comorbidities such as anxiety and depression are significantly more prevalent in patients with IBS than in healthy individuals, underscoring the role of neurogastrointestinal interaction disorders in its pathophysiology (5). While the Rome IV criteria have optimized the diagnostic framework through symptom cluster classification, current pathophysiological models highlight the interaction of multiple mechanisms, including dysregulation of the brain–gut axis, an imbalance of the gut microbiome, and visceral hypersensitivity (6, 7). As a result, the use of traditional treatment approaches, such as antispasmodics, microecological modulators, and cognitive behavioral interventions, is often limited in clinical practice owing to inadequate evidence to support their use and poor long-term adherence (8).

Although pharmacological interventions such as anticholinergics and neuromodulators can partially relieve the symptoms of IBS, their overall clinical efficacy remains modest and is further complicated by a placebo effect of up to 37.5%, posing methodological challenges in accurate assessment of effectiveness (9, 10). Notably, neuromodulators like tricyclic antidepressants and serotonin–norepinephrine reuptake inhibitors are often associated with adverse effects, including nausea, vertigo, and sleep disturbances, which significantly impact adherence with treatment (9). Furthermore, most neuromodulators, including selective serotonin reuptake inhibitors and antiepileptic agents, are used off-label for IBS (11). Clinical survey data from the USA show that fewer than 25% of patients with IBS achieve complete remission of symptoms (12).

However, a Cochrane review (13) has highlighted significant limitations and heterogeneity in existing trials. Our review extends this work by focusing specifically on the comparative effects of structured exercise programs versus non-exercise controls on three key patient-centered outcomes: IBS symptom severity, disease-specific quality of life, and anxiety symptoms.

Therefore, this systematic review and meta-analysis primarily aim to assess the clinical effects of structured exercise therapy by:

1. Synthesizing evidence on IBS symptom severity and health-related quality of life.
2. Evaluating its impact on anxiety symptoms in patients with IBS.

By integrating evidence up to February 2025, this study builds on existing reviews while further focusing on the simultaneous assessment of multiple clinical outcomes (symptoms, quality of life, anxiety) and aims to explore the sources of heterogeneity in exercise programs and population characteristics. We hope that through this analysis, we can not only validate the overall effects of exercise interventions but also provide direct evidence for developing personalized exercise prescriptions and promoting precision management of IBS.

## Methods

### Protocol registration

This systematic review and meta-analysis was registered on PROSPERO under the registration number CRD420250478248. The study was conducted in strict accordance with the Preferred Reporting Items for Systematic Reviews and Meta-Analyses (PRISMA) statement and the Cochrane Handbook for Systematic Reviews and Meta-Analyses.

### Literature retrieval

We systematically searched the PubMed, Embase, Web of Science, and Ovid electronic databases up to February 17, 2025. The complete, reproducible search strategies for all databases are provided in Table 1.

**Table 1:** Complete and reproducible electronic search strategies (aligned with PROSPERO protocol CRD420250478248).

Database	Number	Search strategy
PubMed	–	((exercise[Title/Abstract]) OR (exercise[MeSH Major Topic]) OR (yoga[Title/Abstract]) OR (Tai Chi[Title/Abstract]) OR (physical activity[Title/Abstract])) AND ((Irritable Bowel Syndrome[MeSH Major Topic]) OR (IBS[Title/Abstract]) AND (clinical trial[Filter])
WOS	#1	(TI=(exercise) OR AB=(exercise) OR AB=(yoga) OR TI=(yoga) OR TI=(Tai Chi) OR AB=(Tai Chi)) and Preprint Citation Index (Exclude – Database)
	#2	TS=(Irritable Bowel Syndrome) OR TS=(IBS) and Preprint Citation Index (Exclude – Database)
	#3	#2 AND #1 and Preprint Citation Index (Exclude – Database)
Ovid	1	exercise.ab. or exercise.ti. or yoga.ab. or yoga.ti. or physical activity.ab. or physical activity.ti. or tai chi.ab. or tai chi.ti.
	2	irritable bowel syndrome.ab. or irritable bowel syndrome.ti. or ibs.ab. or ibs.ti.
	3	clinical trial.af.
	4	1 and 2 and 3
Embase	#1	'exercise'/exp/mj OR exercise:ab,ti OR yoga:ab,ti OR 'tai chi':ab,ti
	#2	'irritable bowel syndrome'/exp/mj OR 'irritable bowel syndrome':ab,ti OR ibs:ab,ti
	#3	#1 AND #2

### Research screening criteria

**Figure 1:**
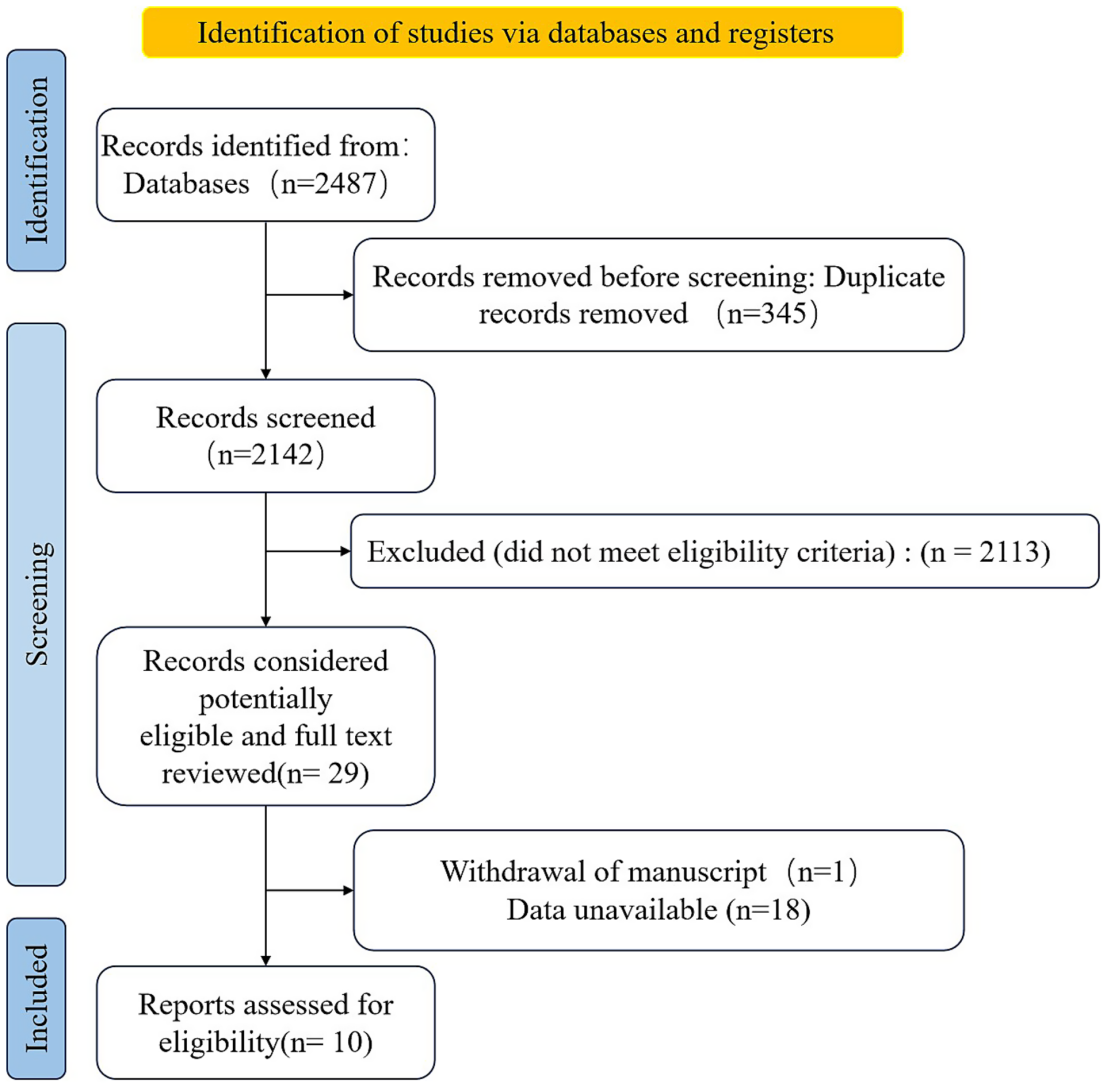
Preferred Reporting Items for Systematic Reviews and Meta-Analyses (PRISMA) diagram analysis showing search results for meta-analysis.

The literature screening process is shown in Figure 1. Duplicates were automatically identified and removed using EndNote X9 literature management software. Cross-library duplicates, multilingual versions, and staged research reports were manually identified by two researchers (Jiali Wu, Shaojie Du) working independently. Double-blind screening was performed initially based on title and abstract, focusing on type of study (e.g., randomized controlled trial [RCT]), intervention (exercise therapy/usual care), and correlation of outcome indicators.

The following inclusion criteria were applied: RCTs published in English (a restriction imposed for feasibility of accurate data extraction and assessment) with no patient age or sex limitations; diagnosis of IBS clearly meeting the Rome I–IV criteria (14–17) and inclusion of an intervention group that received a structured exercise program (with definition of type, frequency, and course of treatment) and a control group that received conventional drugs or basic lifestyle guidance. The exclusion criteria were as follows: RCT of exercise therapy including both an intervention group and a control group; original data missing, and the author could not be contacted; duplicate reports for the same data set; and a non-randomized controlled experimental design.

### Data extraction and integration

The data were collected in a double-blind manner by two independent researchers (Jiali Wu, Yanting Sun) using a pre-validated data extraction table. The extraction table included study characteristics (first author, publication year, country, study design), baseline information, including demographic characteristics (age, sex), movement parameters (type, cycle), and outcome measures (IBS Symptom Severity Scoring System [IBS-SSS], Irritable Bowel Syndrome Quality of Life measure [IBS-QOL], and anxiety score).

Subgroup data pooling was used whereby multi-exercise subgroups (e.g., aerobic exercise, core training) were combined using an inverse variance weighted method. Subgroup A (sample size, N; mean, M; standard deviation, SD) and subgroup B (N, M, SD) were combined using the following equation:

$$\begin{array}{c} N=N_{1}+N_{2}\\ M=\frac{N_{1}M_{1}+N_{2}M_{2}}{N}\\ \mathrm{SD}=\sqrt{\frac{(N_{1}-1)SD_{1}^{2}+(N_{2}-1)SD_{2}^{2}+\frac{N_{1}N_{2}}{N}{\left(M_{1}-M_{2}\right)}^{2}}{N-1}} \end{array}$$

If data from multiple subgroups needed to be combined, the data from the two subgroups could be combined first, and then the obtained data could be combined with the third subgroup using the above formula. Disagreements between reviewers were resolved by consensus or consultation with a third reviewer (Shaojie Du).

### Deviation risk and quality assessment

Two reviewers used Review Manager 5.3 to independently assess the risk of bias of the included studies, based on the following: random sequence generation (selection bias), assignment hiding (selection bias), subject and person blindness (implementation bias), outcome assessment blindness (detection bias), incomplete outcomes data (loss of follow-up bias), selective reporting (reporting bias), and other potential sources of bias. Disagreements were resolved by discussion. The included trials were classified as low quality, high quality, or medium quality according to the following criteria: considered low quality if randomization or assignment concealment was assessed as having a high risk of bias, regardless of the risk of other items; considered high quality when randomization and assignment concealment were assessed as having a low risk of bias and all other items as having a low or unclear risk of bias; and did not meet the high or low risk criteria and considered to be of moderate quality.

### Statistical analysis

The statistical analysis was performed using Review Manager 5.3 software. The effect of exercise intervention on the outcome variables was estimated by comparing the pooled mean difference (MD) and SD of changes before and after treatment between the exercise group and the control group. Continuous data are presented as the MD with the 95% confidence interval (CI). We assessed heterogeneity by visual inspection of forest plots and by the I^2^ statistic. The I^2^ test determines whether there is significant heterogeneity. According to the Cochrane manual (18), the I^2^ statistic is interpreted as follows: 0–25%, low heterogeneity; 25–50%, moderate heterogeneity; and >50%, significant heterogeneity.

A random effects model was used because of the heterogeneity of the exercise therapy interventions. In the literature included in this study, some trials may have incorporated multiple exercise intervention groups or multiple control groups. Since the objective of this study was to compare the efficacy of exercise versus non-exercise therapies for IBS, we employed the subgroup combination formula to merge multiple exercise arms into a single exercise group, or multiple control arms into a single control group, ensuring each study contributed only one independent comparison to the meta-analysis. To ensure that participants in each independent study contributed only once to the pooled effect size, we adopted the following strategy to avoid double counting: If a study compared two (or more) different types of exercise interventions or different non-exercise intervention control groups, we merged the sample sizes, means, and standard deviations of these groups using the subgroup combination formula into a single group for meta-analysis. The merging method followed the formulas provided in the “Data Extraction and Integration” section above. To ensure the principle of independence, all merging or selection operations were completed during the data extraction phase, guaranteeing that each comparison included in the final meta-analysis was statistically independent.

For studies with large heterogeneity, we used Stata14 software for sensitivity analysis to evaluate the stability of the results.

## Results

### Search results and research options

Using a systematic cross-database search strategy, 2,487 initial study records were obtained from the four major databases. After automatic identification by EndNote X9 software and independent review by two researchers, 345 duplicate publications were systematically eliminated, and the remaining 2,142 articles entered the primary screening stage for titles and abstracts. Based on the preset inclusion/exclusion criteria, 2,113 ineligible studies were excluded after double-blind screening, and 29 articles were finally retained for full-text assessment.

In this full-text review stage, further studies that did not meet the requirements were excluded, including one study for which the manuscript was withdrawn, eight studies that had missing raw data and could not be traced, and 10 studies that did not meet the inclusion or exclusion criteria. Finally, 10 RCTs that met the methodological criteria were included in this systematic review and meta-analysis (Figure 1). The basic characteristics of the included studies and reference coding are detailed in the literature (19–28).

### Features included in the study

We selected 10 RCTs (19–28) that included 437 participants (exercise group, *n* = 215; control group, *n* = 222; Table 2). All studies reported the age of the participants, which ranged from 19 years to 43.5 years. The duration of treatment varied from 4 to 24 weeks. All studies compared an exercise group (including yoga, pilates, treadmill exercise, and walking) and a non-exercising control group (including usual care, medication interventions, and dietary recommendations). There was a large deviation in the intensity of self-reported pain between the exercise and control baseline values included in one RCT (23). No studies reported cost-effectiveness, number of recurrent episodes, or adverse events.

**Table 2:** Features of included studies.

**PART 1**															
**Number**	**Records**	**Title**	**Treatment**	**Sample size**			**Baseline**								
				**Exercise group**	**Control group1**	**Control group2**	**Exercise group**			**Control group1**			**Control group2**		
							**Age**	**Male**	**Female**	**Age**	**Male**	**Female**	**Age**	**Male**	**Female**
1	Daley et al. (19)	The Effects of Exercise upon Symptoms and Quality of Life in Patients Diagnosed with Irritable Bowel Syndrome: A Randomised Controlled Trial	exercise	28	28	_	43.1	6	22	43.1	9	19	_	_	_
2	D'Silva et al. (20)	Meditation and Yoga for Irritable Bowel Syndrome: A Randomized Clinical Trial	yoga	38	41	_	43.5	2	36	47.1	37	_	_	_	_
3	Allam et al. (21)	Effect of Pilates exercises on symptoms of irritable bowel syndrome in women: a randomized controlled trial	pilates	30	30	_	29.4	0	30	30.33	0	30	_	_	_
4	Taneja et al. (22)	Yogic Versus Conventional Treatment in Diarrhea-Predominant Irritable Bowel Syndrome: A Randomized Control Study	yogic	9	12	_	30.9	9	0	30.9	12	0	_	_	_
5	Kuttner et al. (23)	A randomized trial of yoga for adolescents with irritable bowel syndrome	yoga	14	11	_	14.32	2	12	13.83	3	8	_	_	_
6	Fani et al. (24)	The effect of aerobic exercises among women with mild and moderate irritable bowel syndrome: A pilot study	treadmill exercise	10	10	_	29.1	0	10	32.7	0	10	_	_	_
7	Evans et al. (25)	Iyengar Yoga for Adolescents and Young Adults With Irritable Bowel Syndrome	yoga	29	22	_	19	2	27	19	6	16	_	_	_
8	Tavakoli et al. (26)	Comparison of Laughter Yoga and Anti-Anxiety Medication on Anxiety and Gastrointestinal Symptoms of Patients with Irritable Bowel Syndrome	laughter yoga	19	18	_	33.1	_	_	31.72	_	_	_	_	_
9	Zhao et al. (27)	Effect of cognitive behavior therapy combined with exercise intervention on the cognitive bias and coping styles of diarrheapredominant irritable bowel syndrome patients	baduanjin	28	29	_	33.75	7	21	36.86	7	22	_	_	_
10	Chao et al. (28)	Interplay of yoga, physical activity, and probiotics in irritable bowel syndrome management: A double-blind randomized study	yoga	10	10	11	36.5	3	7	39.8	4	6	37.9	3	8

**PART 2**							
**Number**	**Records**	**Title**	**Follow-up time**	**Intervention program**			**Outcome measure**
				**Treatment group**	**Control group1**	**Control group2**	
1	Daley et al. (19)	The Effects of Exercise upon Symptoms and Quality of Life in Patients Diagnosed with Irritable Bowel Syndrome: A Randomised Controlled Trial	12 weeks	exercise counseling + pedometer tracking (30 min/day, 5x/week, walking-focused)	usual care	_	IBS-QOL,The Birmingham IBS Symptom Questionnaire,Perceived Stress Scale,The Godin Leisure-Time Exercise Questionnaire
2	D'Silva et al. (20)	Meditation and Yoga for Irritable Bowel Syndrome: A Randomized Clinical Trial	8 weeks	8-week online yoga program with weekly classes (asanas, breathing, meditation) and daily home practice using videos and journals	only receive a 10-minute video education with IBS related resources and information	_	IBS-SSS,IBS-QOL,GAD-7,PHQ-9,MFIS-21,PHQ-15,Perceived Stress Scale, COVID-19 Stress Scale,Self-Compassion Scale-Short Form
3	Allam et al. (21)	Effect of Pilates exercises on symptoms of irritable bowel syndrome in women: a randomized controlled trial	8 weeks	8-week Pilates program (45-50 mins, twice weekly) with dietary advice for the trial group.	dietary advice	_	IBS-SSS, the frequency of complete spontaneous bowel movements,MFIS,HADS,BW
4	Taneja et al. (22)	Yogic Versus Conventional Treatment in Diarrhea-Predominant Irritable Bowel Syndrome: A Randomized Control Study	2 months	2-month yoga intervention (12 asanas + Surya Nadi Pranayama, twice daily)	usual medication, Loperamide 2-6 mg/ day	_	Bowel Symptom Score,Autonomic Symptom Score,STAI,EGG,Parasympathetic Reactivity,Physical Flexibility
5	Kuttner et al. (23)	A randomized trial of yoga for adolescents with irritable bowel syndrome	4 weeks	yoga intervention (including 1 hour of yoga instruction, demonstration and practice, followed by daily video-guided yoga practice at home for 4 weeks)	without any intervention	_	Gastrointestinal Symptoms,FDI,PCQ,RCMAS
6	Fani et al. (24)	The effect of aerobic exercises among women with mild and moderate irritable bowel syndrome: A pilot study	6 weeks	treadmill exercises three times a week for six weeks	continued their usual daily activities.	_	IBS-SSS,IBS-QOL
7	Evans et al. (25)	Iyengar Yoga for Adolescents and Young Adults With Irritable Bowel Syndrome	6 weeks	Iyengar Yoga (1.5 hours twice a week for 6 weeks)	usual care	_	GIS,NRS,CSI,SF-36,FDI,BSI-18,FACIT-Fatigue,PSQI
8	Tavakoli et al. (26)	Comparison of Laughter Yoga and Anti-Anxiety Medication on Anxiety and Gastrointestinal Symptoms of Patients with Irritable Bowel Syndrome	7 weeks	laughter yoga (once a week for 7 weeks)	routine symptom treatment	_	IBS-SSS,BAI
9	Zhao et al. (27)	Effect of cognitive behavior therapy combined with exercise intervention on the cognitive bias and coping styles of diarrheapredominant irritable bowel syndrome patients	24 weeks	24-week CBT combined with Baduanjin exercise	regular medication	_	IBS-SSS,ATQ, DAS, and CSQ
10	Chao et al. (28)	Interplay of yoga, physical activity, and probiotics in irritable bowel syndrome management: A double-blind randomized study	6 weeks	6-week online yoga (40-min sessions, 3x/week) + probiotic supplementation (twice daily, empty stomach)	online yoga + placebo (matched starch capsules) for 6 weeks	6-week probiotic-only intervention	IBS-QOL,BSRS-5,IPAQ,Sit-and-Reach Test,Sit-Up Test,3-Minute Step Test,Gut Microbiome Analysis

### Risk of bias and quality of evidence

**Figure 2:**
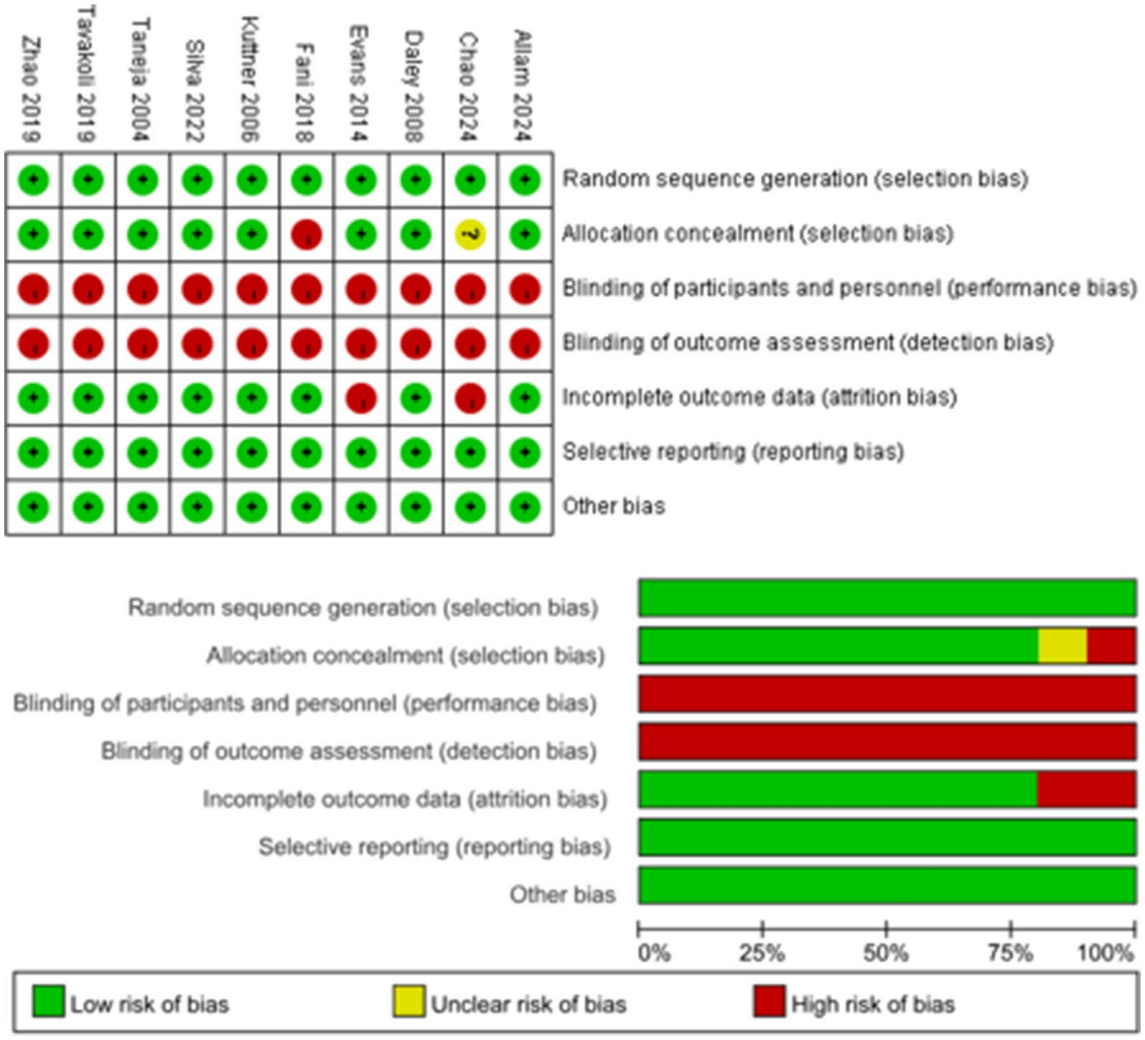
Diagram showing the results of analysis of risk of bias.

Analysis of the quality of the included studies based on the Cochrane Risk of Bias Assessment Tool (RoB 2.0) showed that all trials used random number tables or statistical software for random sequence generation and were assessed as having a low risk of bias. However, one study (28) was judged to be at moderate risk of allocation concealment because the allocation process using opaque envelopes was not described in detail. Another study (24) used the rolling dice odd and even grouping method for allocation concealment and had a high risk of bias. All trials were at high risk at the level of subject blinding and outcome assessment because of the physical limitations of exercise interventions, but none of the 10 trials had subject dropouts and fully reported pre-specified outcomes. Therefore, the risk of loss to follow-up bias and selective reporting bias were low (Figure 2).

### Irritable bowel syndrome-symptom severity score

**Figure 3:**
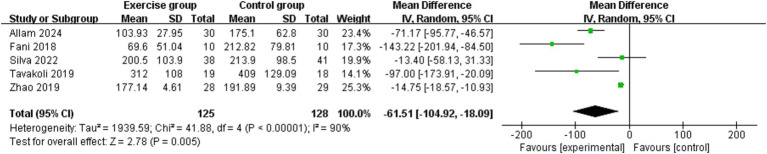
Direct pairwise random-effects meta-analysis of IBS Symptom Severity Scoring System scores.

**Figure 4:**
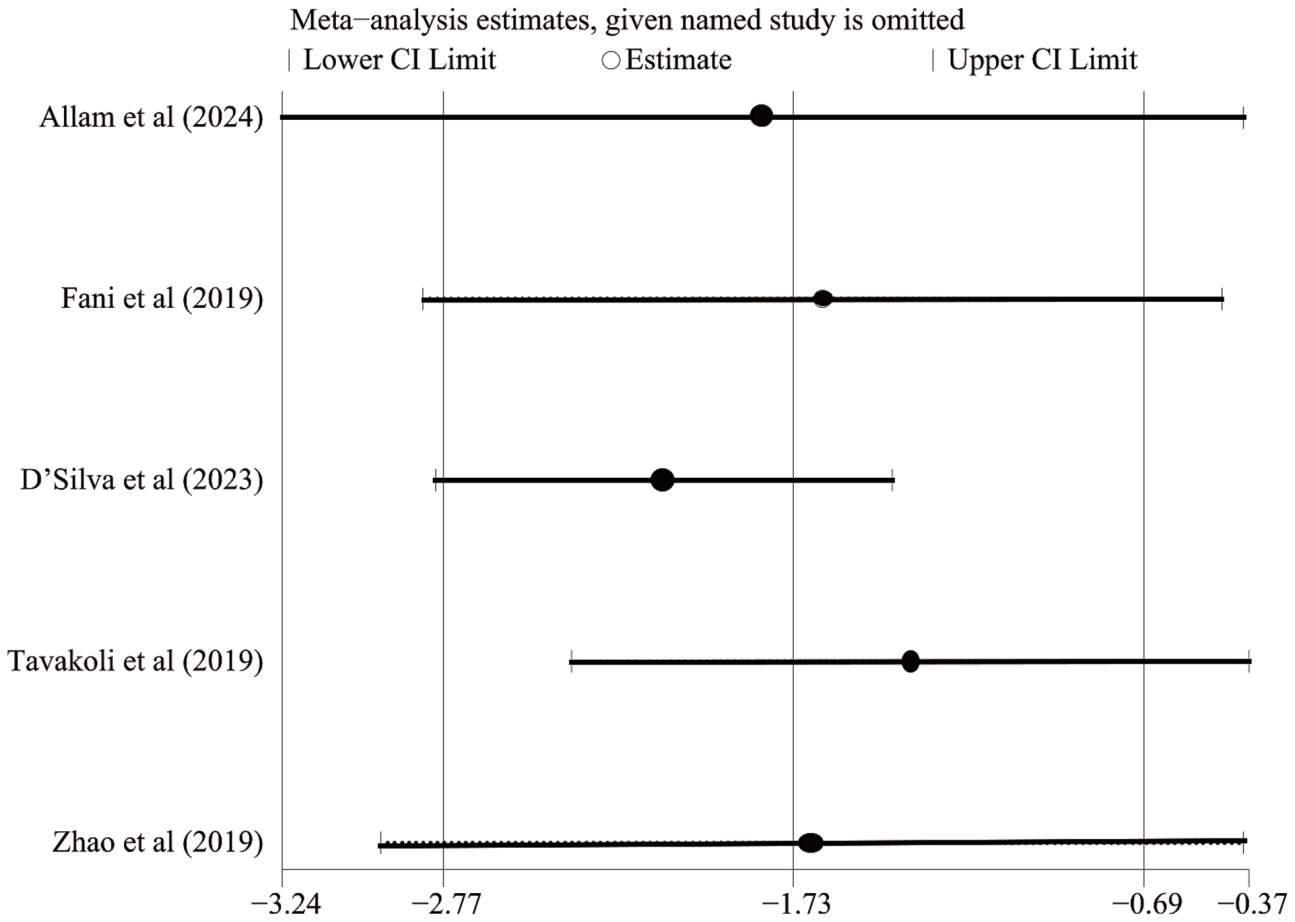
Sensitivity analysis of IBS Symptom Severity Scoring System scores.

We performed a meta-analysis of five RCTs (21, 22, 24, 26, 27) that compared the effects of exercise and non-exercise interventions on symptom severity in IBS (Figure 3). The IBS-SSS was used as the core evaluation tool and assessed the gastrointestinal symptom load by quantifying the comprehensive score (0–500 points) of four dimensions, including intensity of abdominal pain, degree of abdominal distension, frequency of bowel movements and abnormalities, and impairment of daily function during the 10-day observation period (29). The forest plot showed that the test group had a statistically significant advantage in terms of being able to reduce the IBS-SSS score in comparison with the control group (Z = 8.77, *p* < 0.00001). This finding suggested that exercise intervention was effective in relieving the clinical severity of the core symptoms of IBS. However, high heterogeneity was observed (*p* < 0.00001, Cochrane Q test; I^2^ = 90%). Sensitivity analysis confirmed the robustness of this finding, with the effect direction and significance remaining stable after sequential study removal (Figure 4).

### Irritable bowel syndrome quality of life scores

**Figure 5:**
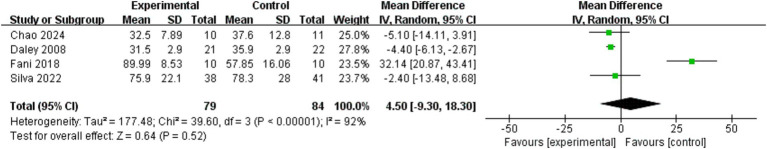
Direct pairwise random-effects meta-analyses of IBS-QOL.

**Figure 6:**
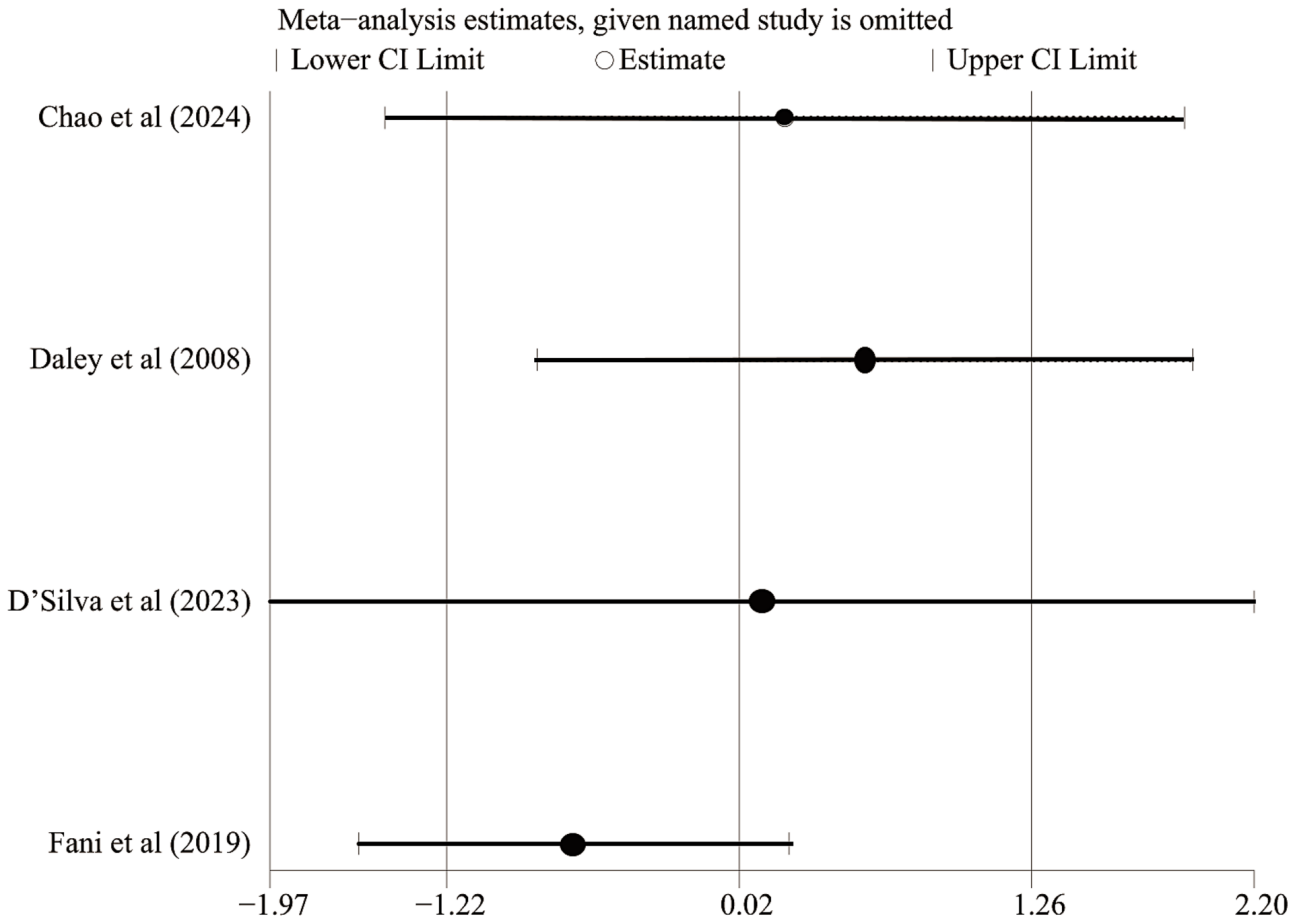
Sensitivity analysis of IBS-QOL.

Meta-analysis of four RCTs (19, 20, 24, 28) was performed to investigate the effect of exercise intervention on quality of life in patients with IBS (Figure 5). The IBS-QOL was used as an assessment measure. This tool covers 34 items in eight dimensions, including emotional distress, limitation of daily function, somatic imagery, health anxiety, dietary avoidance, social adaptation, sexual function, and interpersonal relationships. The scores were standardized and converted to a 0–100-point scale, with increased scores indicating improved quality of life (30). Combined analysis showed no statistically significant difference between the exercise and control groups (*p* = 0.52). However, the results of each study showed significant heterogeneity (I^2^ = 92%, *p* < 0.001). Fani et al. (24) reported that exercise intervention significantly improved IBS-QOL scores (weighted mean difference [WMD] 32.14, 95% CI 20.87–43.41), while Daley et al. (19) observed a negative effect (WMD –4.40, 95% CI –6.13, −2.67). Improvement in IBS-QOL scores did not reach statistical significance in the studies reported by Chao et al. (28) (WMD –5.10, 95% CI –14.11, 3.91) and D’Silva et al. (20) (WMD –2.40, 95% CI –13.48, 8.68). For quality of life (IBS-QOL), the pooled analysis showed no statistically significant difference between groups (*p* = 0.52), despite high heterogeneity (I^2^ = 92%, *p* < 0.001). Sensitivity analysis indicated this non-significant result was not driven by any single study (Figure 6). In summary, the current evidence neither confirms definitive benefits of exercise for IBS-QOL nor rules out its potential efficacy in specific populations or with particular protocols. Therefore, based on the current heterogeneous evidence, no significant benefit of exercise on IBS-specific quality of life can be concluded.

### Anxiety

**Figure 7:**
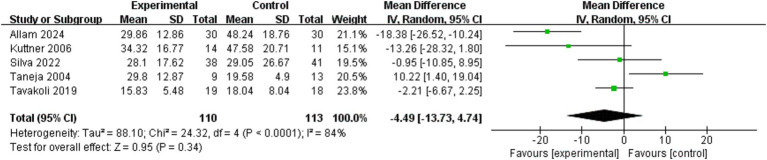
Direct pairwise random-effects meta-analyses of anxiety.

**Figure 8:**
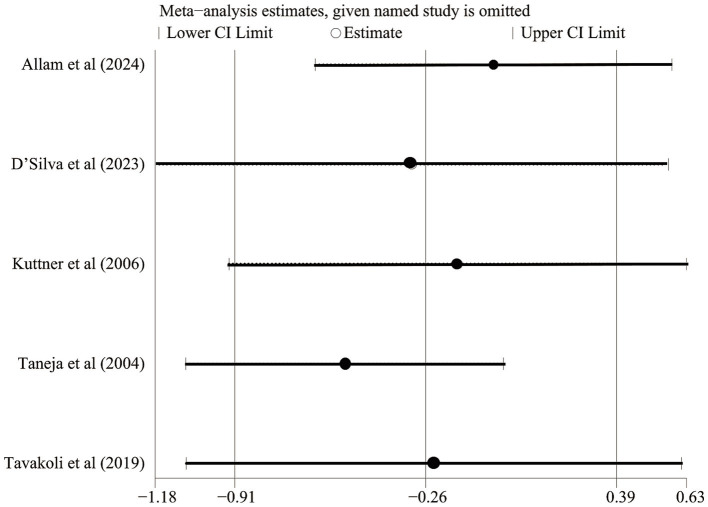
Sensitive analysis of anxiety.

We performed a meta-analysis of the efficacy of exercise intervention in improving anxiety in patients with IBS based on standardized transformed anxiety score data (Figure 7). Given that there is heterogeneity in the anxiety assessment tools described in the original literature (e.g., the Hamilton Anxiety Rating Scale and Hospital Anxiety and Depression Scale), we consistently standardized each scale score to a range of 1–100 by isometric scaling (with a decrease in score suggesting relief of anxiety) and included five studies that met the criteria (20–23, 26). Analysis using a random effects model showed that the overall WMD between the exercise intervention group and the control group was −4.49 points (95% CI –13.73, 4.74); the difference did not reach statistical significance (*p* = 0.34). Allam et al. (21) and Taneja et al. (22) showed that exercise significantly reduced anxiety scores; in contrast, the results reported by Kuttner et al. (23), D’Silva et al. (20), and Tavakoli et al. (26) were not statistically significant. High heterogeneity was observed (I^2^ = 84%, *p* < 0.001). Sensitivity analyses, including one restricted to common anxiety scales, confirmed the non-significant result (Figure 8). Thus, no statistically significant effect of exercise on anxiety was demonstrated. Future studies employing standardized assessment tools and intervention protocols are needed to clarify this issue.

## Discussion

### Potential mechanisms and context for future research

The following sections discuss potential biological mechanisms through which exercise might benefit IBS, as suggested by preclinical and translational studies. These mechanisms are not directly evaluated by the clinical data in this review but provide context for interpreting the findings and designing future research.

### Brain–gut axis regulatory pathway

The brain–gut axis is a bidirectional communication pathway that connects the gut microbiome, gastrointestinal tract, and peripheral and central nervous systems through the vagus-mediated autonomic nervous system (ANS) (31), and dysfunction in this system is considered to be one of the main causes of IBS (32). This regulatory network achieves signal integration through multiple pathways, including vagally mediated bidirectional signaling in the ANS (afferent versus efferent) and neuroendocrine regulation of the hypothalamic–pituitary–adrenal axis (HPA) axis and serotonin system (33–37).

### Remodeling of autonomic balance

IBS is considered a biopsychosocial model and is a product of a stressful environment (38). Pressure usually induces sympathetic activation and suppresses the vagus nerve while activating the sacral parasympathetic nervous system (39). Chronic stress can lead to disruption of the balance of the ANS, such as low vagal tone or high sympathetic tone, which favors pro-inflammatory conditions (9). Imbalances in complex interactions between events occurring in the intestinal lumen (including the gut microbiota), gut mucosa, enteric nervous system, and central nervous system can lead to abnormal gut motility and visceral hypersensitivity (40). Exercise may restore autonomic balance by the following mechanisms.

#### Vagus activation

As part of the parasympathetic nervous system, the vagus nerve has multiple physiological functions, including regulation of immune responses, digestive processes, and the heart rate, and, more recently, control of emotions (41). In a basic study, carbon fiber microelectrodes were used to record the vagal preganglionic neuron population and the vagal dorsal motor nucleus of the brainstem in rats. It was found that the preganglionic neurons of the nucleus ambiguus and the vagal dorsal motor nucleus were strongly activated during exercise. Exercise training significantly increased the resting activity of vagal preganglionic neurons and enhanced the excitatory response of neurons in the nucleus ambiguus during exercise. The investigators concluded that exercise increased the activity of vagal preganglionic neurons, thereby increasing vagal tone (42). The vagus nerve is a key component of the neuroendocrine immune axis and has anti-inflammatory properties; its afferent signals activate the HPA axis and release corticosteroids through the adrenal gland, and its output signals inhibit the release of pro-inflammatory cytokines such as tumor necrosis factor alpha through cholinergic anti-inflammatory pathways and interaction of acetylcholine with α7 nicotinic receptors in the spleen and intestinal macrophages (9, 43). Disruption of the intestinal barrier leads to an increase in lipopolysaccharide and proinflammatory cytokines, which are important pathomechanisms contributing to abdominal pain in patients with IBS (44, 45).

#### Sympathetic inhibition

A basic study used a novel method to assess autonomic function by measuring fingertip blood flow with continuous wave Doppler ultrasound and found increased sympathetic activity in patients with IBS, suggesting that these abnormalities may be involved in the pathogenesis of IBS (46). Exercise training has been shown to attenuate sympathetic activity in experimental animals (47, 48). It has also been clinically demonstrated in two RCTs (49, 50) that exercise is able to reduce sympathetic overactivation. A systematic review and meta-analysis of 40 intervention studies (1,253 patients) (51) investigated the effects of exercise on muscle sympathetic nerve activity in humans and found that exercise training reduced this activity. That study also performed a meta-regression analysis to confirm a dose–response relationship whereby individuals with higher pre-intervention sympathetic activity showed greater reductions in post-intervention sympathetic activity. Therefore, there is evidence demonstrating that exercise can improve the symptoms of IBS by inhibiting sympathetic activity.

### Regulation of the neurotransmitter system

Signals are transmitted in the gut and central nervous system by a variety of neurotransmitters, among which abnormal metabolism of serotonin and dopamine is closely associated with symptoms of IBS.

#### Serotonin signaling pathway

Alterations in signaling of serotonin secreted by enterochromaffin cells have been associated with IBS (52). However, changes in enterochromaffin cells may be a pathophysiological mechanism leading to symptoms of diarrhea in IBS (53). Intestinal chromaffin cells secrete 90% of the serotonin in the body (54). The released serotonin activates multiple receptors expressed in nociceptive afferents and provokes afferent nerves in submucosal terminals, thereby initiating peristaltic reflexes and promoting intestinal secretion and reducing gastrointestinal terminal sensitivity (55–57). One study also reported that the 5-HIAA/serotonin ratio was within the normal range in patients with symptoms of constipation, but decreased in patients with IBS and diarrhea, suggesting that reuptake of serotonin may be reduced in patients with IBS and diarrhea, whereas those who have IBS with constipation may have impaired serotonin release (58). There is a lack of studies directly demonstrating that exercise therapy improves symptoms of IBS by regulating intestinal serotonin levels. However, a basic study has shown that exercise significantly reduces expression of the serotonin transporter in specific regions of the brain (e.g., the rostral ventromedial medulla) and reduces reuptake of serotonin, thereby increasing serotonin levels in the synaptic cleft (59).

### Downregulation of HPA axis activity

Chronic stress causes elevated cortisol levels by activating the HPA axis, inducing intestinal inflammation and increased permeability. Exercise intervention inhibits overactivation of the HPA axis through several mechanisms.

Many studies have confirmed that dysfunction of the HPA axis is one of the causes of IBS, and that reducing activity in the HPA axis is an effective way to improve IBS (60–67). Dysfunction of the HPA axis results in enhancement of the intestinal stress response (64, 68, 69). Studies have reported that elevated basal cortisol levels are a common pathophysiological feature in patients with IBS (63, 70, 71), which may explain the dysregulation of activity in the HPA axis. Exercise of varying intensity has been shown to have different effects on the response of the HPA axis to acute stress, and repeated low-intensity exercise has been shown to reduce cortisol levels, thereby regulating activity in the HPA axis and improving symptoms of IBS (72, 73), while high-intensity exercise causes a proportional increase in cortisol, which may worsen the symptoms of IBS (74). The critical intensity level leading to release of cortisol is about 60% of VO_2max_, and the greater the intensity of exercise, the greater its release (74) and the greater the activation of the HPA axis (37). Perhaps this explains why yoga, as well as activities that involve slow and usually non-sustained activity, can improve symptoms of IBS (75), whereas strenuous exercise leads to increased intestinal permeability, gastrointestinal damage, and mild endotoxemia (76–78).

### Microbiome–gut–brain axis

The brain–gut microbial axis is a bidirectional communication network formed by the gut microbiota, gut, and brain through neurological, endocrine, and metabolic pathways. Its core mechanisms include transmission of vagal signals, regulation of the HPA axis, and the action of microbial metabolites (e.g., short-chain fatty acids [SCFAs]). Key microbiota such as Firmicutes and *Akkermansia muciniphila* enhance intestinal barrier function, inhibit inflammation, and regulate neurotransmitters (e.g., serotonin) and brain-derived neurotrophic factor by producing SCFAs, thereby affecting cognition, mood, and metabolism (33–37). Dysregulation of the gut microbiota is an important mechanism in the pathogenesis of IBS, and its diversity is significantly reduced in patients with IBS (79–81). One study showed that dysbacteriosis of the intestinal flora occurred in 73% of patients with IBS and in only 16% of healthy individuals (82).

Clinical interventions have shown that supplementation with probiotics such as *Bifidobacterium longum* significantly improves intestinal symptoms and mood in patients with IBS by modulating the structure of the microbiota and restoring levels of SCFAs (83–85). However, exercise interventions (especially aerobic exercise) indirectly alleviate inflammation and intestinal dysfunction by increasing the abundance of beneficial bacteria, such as Akkermansia (37). Studies have shown that different types of exercise have significantly different regulatory effects: long-term aerobic exercise (e.g., running or riding) improves intestinal barrier and brain function by increasing the abundance of Firmicutes and SCFA levels (86), while high-intensity exercise (e.g., running a marathon) increases the abundance of Actinobacteria and improves lipid metabolism (87). It is important to note that involuntary exercise (e.g., autonomous running wheels) may not achieve the desired effect by increasing the genera Lactobacillus and *Blautia coccoides* and *Eubacterium rectale* from the phylum Firmicutes, as well as Bifidobacterium from the phylum Actinobacteria (88), as well as voluntary exercise (37). In summary, the reviewed evidence suggests exercise could represent a new strategy for intervening in IBS by optimizing the structure of the microbiota and its functional metabolism in multiple dimensions.

### Analysis of results

In this meta-analysis, exercise therapy significantly improved IBS symptom severity (IBS-SSS; *p* < 0.00001) but did not show statistically significant effects on disease-specific quality of life (IBS-QOL) or anxiety symptoms. However, this result must be interpreted with caution due to high heterogeneity (I^2^ = 84–92%), the small number of trials (*n* = 10), and their limited sample sizes. The source of heterogeneity can be attributed to multiple dimensions. High heterogeneity primarily stems from differences in intervention protocols and study populations, as well as methodological design inconsistencies across studies. The first is the heterogeneity of intervention regimens. The types of exercises used in the various studies (e.g., yoga, Pilates, aerobic exercise) differ significantly in terms of intensity parameters, with high-intensity exercise partially counteracting the improvement of anxiety by activating the HPA axis, leading to increased cortisol levels, while low-intensity intervention has limited efficacy because it does not reach the neurotransmitter release threshold. The second source is population baseline heterogeneity, where too high a proportion of women were included in the study, sex-related hormonal fluctuations (e.g., estrogen modulation of gut sensitivity) were not adjusted for, and patients were not stratified by Rome IV subtype (e.g., IBS patients with diarrhea had a higher baseline sympathetic tone and may be more sensitive to exercise intervention). The third source was measurement tool and assessment bias. In quality of life assessment, complex dimensional improvements such as “social functioning” (e.g., the study by D’Silva et al.) and “sexual health” lagged behind somatic symptoms, while sensitivity differences on anxiety scales (e.g., the study by Taneja et al. detected significant improvements using STAI, while Kuttner et al. found no differences using the Revised Children’s Manifest Anxiety Scale) and exercise interventions could not be implemented blindly to further weaken the significance of differences between groups. IBS-QOL did not improve or was associated with a “time window effect” of brain-gut axis signal integration, while bidirectional regulation of anxiety states (low intensity inhibition vs. high intensity activation of the HPA axis) and individual differences in the brain–gut–microbiota axis suggest the need for individualized intervention strategies. Most importantly, the high heterogeneity across outcomes means that the pooled estimates should be interpreted with caution, as they derive from studies differing substantially in interventions, populations, and measures, which limits the reliability and generalizability of the conclusions.

### Shortcomings and prospects

The current evidence system has four levels of limitations. The first is methodological shortcomings: the average sample size of the included studies is too low, the statistical power is insufficient, and most of the studies do not use objective quantitative indicators, resulting in difficulty in modeling the exercise dose–response relationship; at the same time, the heterogeneity of anxiety assessment tools triggers effect dilution and scale focus differences, and threshold criteria are different, further interfering with the integration of results. The second limitation is blank mechanism analysis, whereby some studies lack multiple groups of student markers (plasma brain-derived neurotrophic factor, fecal 5-HIAA, intestinal microbiota, SCFAs) and cannot elucidate the mechanism of motor–microbiota–brain axis interaction; in particular, the temporal association between vagally mediated cholinergic anti-inflammatory pathways and serotonin reuptake regulation has not been quantified. The third limitation is the barrier to clinical translation, in that the longest follow-up period is only 6 months, and cost-effectiveness analysis and safety data are missing, limiting health decision-making support. Another important limitation of this review is the potential introduction of publication language bias. To ensure the accuracy of data extraction and risk of bias assessment, we only included studies published in English. Although English is the predominant language of international medical research, this restriction may still have led us to overlook high-quality evidence in other languages (such as Chinese), potentially affecting the generalizability and effect estimates of the summary results. In future studies, we will expand the scope of languages to ensure the inclusion of high-quality literature from other languages, thereby providing higher-level clinical evidence for exercise therapy interventions in IBS.

Future research could explore several promising directions. The first is to investigate precision prescriptions: for example, future trials could test whether a targeting regimen based on Rome IV subtype was developed, namely, IBS-C used core muscle training (3 times a week, 60% 1RM load) combined with abdominal breathing (6 times/min) to enhance colon propulsive force, IBS-D implemented low-impact aerobic exercise (40–55% heart rate reserve) combined with vagal activation (expiratory/inspiratory ratio 2:1). The second strategy is to develop multi- modal evaluation systems, which might integrate resting brain functional MRI, metagenomic sequencing, and dynamic HRV monitoring with the aim of building a machine learning-driven efficacy prediction model (area under the curve ≥0.85). The third strategy is to optimize mixed interventions, potentially using a 2 × 2 factorial design to explore the synergistic effect of exercise with probiotics and neural regulation, and a closed-loop adaptation system was developed. Finally, a long-term goal would be the deep integration of evidence- based medicine and precision medicine, potentially promoting exercise therapy from an “auxiliary intervention” toward a core module of stepped care for IBS, with the aim of achieving a triple optimization of symptom control, functional recovery, and health economic benefit.

## Conclusion

This systematic review and meta-analysis of 10 RCTs provides low-certainty evidence that exercise therapy may reduce symptom severity in IBS, primarily due to high heterogeneity and imprecision. Meta-analysis based on a random-effects model showed statistically significant differences between exercise interventions in improving IBS core symptoms, suggesting that it may exert therapeutic effects by modulating gut motility, visceral sensitivity, or neuroendocrine pathways. However, combined analysis of health-related quality of life (using the IBS-QOL scale) and anxiety failed to obtain statistically significant evidence, suggesting that there may be intervention type-specific or threshold effects of exercise therapy on multidimensional health outcomes.

Our safety analysis showed that no serious adverse events directly related to exercise intervention were reported in the included studies, confirming their safety in short-term application. However, it is important to point out that the current evidence has significant methodological limitations: (1) high between-study heterogeneity (I^2^ = 84–92%), mainly owing to the heterogeneity of intervention regimens (yoga, aerobic exercise, and other pattern differences), treatment span (4–24 weeks), and baseline characteristics of the population (sex, age, symptom subtypes); (2) multiplicity of measurement tools for subjective outcome measures (e.g., quality of life, anxiety) and lack of blinding may lead to amplification of bias; and (3) long-term effects and dose–response relationships have not been clearly established.

In summary, exercise therapy as an adjunct intervention for IBS has potential clinical value for symptom relief and the advantages of non-invasiveness and low cost, but its ability to improve multidimensional health outcomes needs to be further verified by high-quality RCTs. Future large-scale, well-designed RCTs with standardized interventions are needed to confirm these clinical effects. If benefits are confirmed, such trials could incorporate mechanistic evaluations (e.g., of autonomic function, microbiota, or neuroendocrine markers) to elucidate the pathways involved.

## Data Availability

The original contributions presented in the study are included in the article/Supplementary material, further inquiries can be directed to the corresponding authors.
