# Supplementary material for: Evaluation of the effectiveness of exercise therapy for irritable bowel syndrome: a systematic review and meta-analysis
Source: Front Med (Lausanne). 2026 Mar 10;13:1771521. doi: 10.3389/fmed.2026.1771521 (PMC13010347; doi:10.3389/fmed.2026.1771521)
Supplement: Supplementary file 1 [file Table_1.docx]

Supplementary Table 1. List of Abbreviations Used in the Manuscript

| Abbreviation | Full Form |
| --- | --- |
| 5-HIAA | 5-Hydroxyindoleacetic Acid |
| ANS | Autonomic Nervous System |
| BDNF | Brain-Derived Neurotrophic Factor |
| BMI | Body Mass Index |
| CI | Confidence Interval |
| CRediT | Contributor Roles Taxonomy |
| DOI | Digital Object Identifier |
| FEM | Fixed-Effects Model |
| HPA | Hypothalamic-Pituitary-Adrenal Axis |
| HRV | Heart Rate Variability |
| IBS | Irritable Bowel Syndrome |
| IBS-C | Irritable Bowel Syndrome with Constipation |
| IBS-D | Irritable Bowel Syndrome with Diarrhea |
| IBS-QOL | Irritable Bowel Syndrome Quality of Life Questionnaire |
| IBS-SSS | Irritable Bowel Syndrome Symptom Severity Score |
| IV | Inverse Variance |
| MD | Mean Difference |
| PRISMA | Preferred Reporting Items for Systematic Reviews and Meta-Analyses |
| PROSPERO | International Prospective Register of Systematic Reviews |
| RCT | Randomized Controlled Trial |
| REM | Random-Effects Model |
| RoB | Risk of Bias |
| SCFA | Short-Chain Fatty Acid |
| SD | Standard Deviation |
| STAI | State-Trait Anxiety Inventory |
| RCMAS | Revised Children's Manifest Anxiety Scale |
| VO₂max | Maximal Oxygen Consumption |
| WMD | Weighted Mean Difference |

Caption:

Supplementary Table 1 | List of abbreviations used in the manuscript.

This table provides the full forms of all abbreviations appearing in the main text, figures, and tables of the article "Effects of Structured Exercise Therapy on Symptom Severity, Quality of Life, and Anxiety in Patients with Irritable Bowel Syndrome: A Systematic Review and Meta-Analysis of Randomized Controlled Trials". The abbreviations are listed in alphabetical order to assist readers in understanding the terminology and technical terms used throughout the article. All abbreviations are defined at first mention in the manuscript, and this supplementary table serves as a quick reference guide.
